# Fecal microbiota transplanted from old mice promotes more colonic inflammation, proliferation, and tumor formation in azoxymethane-treated A/J mice than microbiota originating from young mice

**DOI:** 10.1080/19490976.2023.2288187

**Published:** 2023-11-29

**Authors:** Nicholas A. Crossland, Samuel Beck, Wei Yu Tan, Ming Lo, Joel B. Mason, Chao Zhang, Weimin Guo, Jimmy W. Crott

**Affiliations:** a Department of Pathology and Laboratory Medicine, Boston University Chobanian and Avedisian School of Medicine, Boston, USA; b Comparative Pathology Laboratory, Boston University National Emerging Infectious Disease Laboratories, Boston, MA, USA; c Department of Dermatology, Boston University Chobanian and Avedisian School of Medicine, Boston, MA, USA; d Vitamins and Carcinogenesis Laboratory, Jean Mayer USDA Human Nutrition Research Center on Aging at Tufts University, Boston, MA, USA; e Department of Medicine, Boston University Chobanian and Avedisian School of Medicine, Boston, MA, USA

**Keywords:** Microbiome, fecal microbiota transplant (FMT), aging, colorectal cancer (CRC), mitochondria, inflammation, azoxymethane

## Abstract

Aging is a strong risk factor for colorectal cancer (CRC). It is well established that gut microbial dysbiosis can play a role in the etiology of CRC. Although the composition of the gut microbial community changes with age and is reported to become more pro-inflammatory, it is unclear whether such changes are also pro-tumorigenic for the colon. To address this gap, we conducted fecal microbiota transplants (FMT) from young (DY, ~6 wk) and old (DO, ~72 wk) donor mice into young (8 wk) recipient mice that were pre-treated with antibiotics. After initiating tumorigenesis with azoxymethane, recipients were maintained for 19 wk during which time they received monthly FMT boosters. Compared to recipients of young donors (RY), recipients of old donors (RO) had an approximately 3-fold higher prevalence of histologically confirmed colon tumors (15.8 vs 50%, Chi_2_ *P* = .03), approximately 2-fold higher proliferating colonocytes as well as significantly elevated colonic IL-6, IL-1β and Tnf-α. Transcriptomics analysis of the colonic mucosa revealed a striking upregulation of mitochondria-related genes in the RO mice, a finding corroborated by increased mitochondrial abundance. Amongst the differences in fecal microbiome observed between DY and DO mice, the genera *Ruminoclostridium, Lachnoclostridium* and *Marvinbryantia* were more abundant in DY mice while the genera *Bacteroides* and *Akkermansia* were more abundant in DO mice. Amongst recipients, *Ruminoclostridium* and *Lachnoclostridium* were higher in RY mice while *Bacteroides* was higher in RO mice. Differences in fecal microbiota were observed between young and old mice, some of which persisted upon transplant into recipient mice. Recipients of old donors displayed significantly higher colonic proliferation, inflammation and tumor abundance compared to recipients of young donors. These findings support an etiological role for altered gut microbial communities in the increased risk for CRC with increasing age and establishes that such risk can be transmitted between individuals.

## Introduction

There are approximately 135,000 new cases and 50,000 deaths per year from colorectal cancer (CRC) in the US.^1^ The risk of CRC approximately doubles every decade after the age of 40^2^ or earlier.^3^ Because the population is rapidly aging, with a doubling in the number of citizens over the age of 65 in the next 45 y,^4^ the burden of CRC is predicted to increase substantially.

It is now well established that an imbalance of the gut microbial composition, or dysbiosis, plays an important causal role in the etiology of CRC.^5–16^ Differences between the gut microbiome of individuals with CRC and healthy controls have been described^5,6^ and observations that an altered microbiota is already present among individuals with precancerous adenomas^7–10^ suggest an involvement preceding the appearance of cancer. A causal role of gut microbiota in CRC is supported by reports that stool from tumor-bearing mice^15^ or specific communities from human donors^11^ can promote tumor formation in recipient mice. Moreover, individual species including *F. nucleatum*,^12^ enterotoxigenic *B. fragilis*^13^ and *E. coli NC101*^14^ can also promote tumor formation in mice. Further supporting a role for the gut microbiota in CRC is observations that antibiotic treatment^15^ and germ-free conditions^16^ suppress tumor formation in mice. One mechanism by which gut bacteria promote CRC is by promoting inflammation. Inflammation affects most of the major processes that govern the evolution of a tumor including mutation, proliferation, apoptosis, vascularization, invasion and immune evasion.^17^

The occurrence of chronic low-grade inflammation with aging, characterized by elevated IL-6 and C-reactive protein among others, led to the coining of the term *inflamm-aging*.^18^ Observations that the composition of the gut microbiome changes with age and correlates with various markers of systemic and colonic inflammation^19–21^ and mucin depletion^22^ suggest that gut microbial dysbiosis could be a driver of inflamm-aging. Importantly, Fransen *et al*.^23^ demonstrated that gut bacteria from old mice increased intestinal expression of pro-inflammatory genes, including Tnfα and increased activation of the pro-inflammatory Toll-like receptor 4 (TLR4) signaling when inoculated into young mice – thus demonstrating that an age-altered gut microbiome *per se* is sufficient to create an inflammatory milieu.

Interestingly, the ramifications of transplanting fecal material from old to young mice are not constrained to the gut. Indeed, others report that transplanting fecal microbiota from old to young mice induces various aspects of CNS dysfunction, behavioral abnormalities and cognitive decline^24–27^ as well as increased body fatness and hyperinsulinemia.^28^

Despite robust evidence that age-altered gut microbial communities are pro-inflammatory and can trigger a wide array of physiological disturbances both in the gut and distant sites including the brain, it remains unclear whether such shifts in gut microbial community can promote colon tumor formation. We aimed to address this gap and conducted a study in which fecal material from young and old donor mice was transplanted into young recipient mice which were subsequently challenged with the chemical carcinogen azoxymethane. The proportion of mice receiving FMT from old donors that developed colon tumors (adenoma and adenocarcinoma) was three-fold greater than those receiving FMT from young donors. Concomitant with increased tumor incidence, RO mice displayed an approximately 2-fold higher abundance of proliferative cells in the colon and a significantly higher abundance of colonic IL-6, IL-1β and Tnf-α compared to RY mice. RNA-seq and IHC analyses suggest increased mitochondrial biogenesis in RO mice, which could support tumorigenesis by providing energy for proliferation and increased production of reactive oxygen species. These data are consistent with the hypothesis that age-altered gut microbial communities are pro-tumorigenic for the colon.

## Methods

### Animal study

All procedures and protocols were approved by the Institutional Animal Care and Use Committee of Tufts University, Boston, MA. All animals were individually housed in ventilated cages and maintained on a 12 h light/dark cycle with unrestricted access to chow diet and water.

#### Donor cohort

Mice were purchased from The Jackson Laboratory (Bar Harbor, ME). Young (6 wk. *N* = 10 male + 10 female) and old (72 wk. *N* = 10 male + 10 female) C57BL6/J donor mice were allowed to acclimate for 1 wk and then maintained for another 3–4 wk to allow for daily collection of fecal material. To collect fecal material, each mouse was placed in a clean plastic cage without food or bedding and observed until defecation (usually 5–10 min). Fecal material was removed to a sterile microfuge tube immediately upon production and then frozen in liquid N_2_ before storage at −80°C as previously described.^28^

To assess gut barrier permeability donors were fasted in a clean cage without food or bedding. After 4 h, mice were gavaged with 0.6 mg Fluorescein Isothiocyanate Dextran 3-5kDa (FD4. Sigma, St Lous, MO)/g body weight, and after a further 4 h, blood was collected by cardiac puncture. Donor mice were then euthanized by cervical dislocation. Blood was allowed to clot, and serum stored at −80°C.

#### Recipient cohort

**Figure 1.**
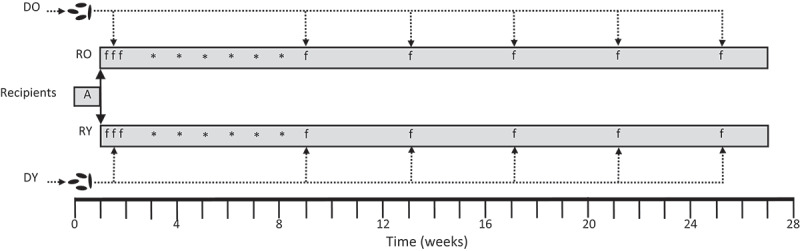
Experimental design.

Eight-week-old A/J mice (*N* = 20 (10 male + 10 female) per group) served as fecal microbiota transplant (FMT) recipients. After acclimation for 10 d, mice were transferred to sterile cages with sterile bedding. Resident gut microbes were depleted by providing an antibiotic cocktail consisting of neomycin (1 g/L, Sigma), vancomycin (0.5 g/L, Cayman. Ann Abor, MI), metronidazole (0.5 mg/L, Sigma) and ciprofloxacin (0.125 g/L, Sigma) in drinking water according to the method of Yu et al.^29^ Sucrose (3 g/L, Sigma) was added to improve the palatability of the cocktail. Drinking water was replaced daily and the cocktail made fresh every 2 d. Antibiotics were provided in this fashion for 7 d. After 1 d of washout on fresh sterile drinking water, recipient mice were randomly assigned to receive FMTs from one specific (sex matched) donor mouse – thus generating 40 donor-recipient pairs (Figure 1).

DY, Young Donor mice (~6 wks. *N* = 20); DO, Old Donor mice (~72 wks. *N* = 20); RY, Recipients of FMT from Young mice (*N* = 20); RO, Recipients of FMT from Old mice (*N* = 20); A, antibiotic cocktail; f, fecal microbiota transplant; * azoxymethane injection (1 at 5 then 5 at 10 mg/kg IP).

To prepare FMT samples, 1–2 frozen fecal pellets were added to 600 µl of anaerobic PBS (containing 0.5 g/L L-cysteine) and then disaggregating using a 1 ml pipette tip with N_2_ gently bubbling through. Samples were then vortexed vigorously for 5–10 s and briefly spun in a microfuge to pellet larger debris. Two-hundred and fifty µl of supernatant was drawn into a 1 ml syringe fitted with flexible disposable gavage needle (Fisher Scientific, Waltham, MA) and administered by gavage to mice lightly sedated with isoflurane. Additional FMTs were conducted 3 and 7 d after the first FMT (with freshly prepared samples).

After 1-wk, mice were given six weekly IP injections with the colon carcinogen azoxymethane (AOM. Sigma) (5 mg/kg for first then 10 mg/kg for remainder) to initiate tumorigenesis. One week after the final AOM injection, mice were given a FMT booster and every 4 wk thereafter for the remainder of the study. Thus, an initial series of three FMTs were conducted in 1 wk to colonize the recipients, and then five monthly boosters were conducted to maintain engraftment (total = 8 FMT per mouse).

Body composition was assessed by MRI (EchoMRI, Houston, TX) 18 wk after the final AOM injection. One week later, mice were anesthetized via isoflurane inhalation and then euthanized by exsanguination via cardiac puncture followed by cervical dislocation. Whole blood was collected into untreated microfuge tubes and allowed to clot for 20 min on ice. Serum was collected after centrifugation (2000 × *g* for 10 min) and stored frozen at −80°C. Colons were removed, opened longitudinally and fecal contents snap frozen in liquid N_2_. Colons were then rinsed thoroughly in ice-cold PBS containing protease and phosphatase inhibitors (Roche, Indianapolis, IN). Colons were then inspected for the presence of tumors by a blinded investigator under a dissecting microscope. After measuring size and location, tumors were excised and fixed in 10% formalin and preserved in 70% ethanol for later histologic review. A 0.5 cm section from the center of the colon, and without any apparent lesions, was cut and fixed in formalin for later IHC analyses. Finally, the mucosa was scraped from the remaining normal-appearing colon with glass microscope slides, and frozen in liquid nitrogen prior to storage at −80°C.

## Tissue analyses

Donor serum FD4 concentrations were measured using a fluorescent plate reader (BMG Labtech, Cary, NC) with excitation 485 nm and emission at 520 nm. Serum endotoxin was measured using the Limulus Amebocyte Lysate assay (Thermo, Waltham, MA). Serum LPS binding protein (LBP) was measured by ELISA (Abcam, Cambridge, MA). Serum IL-6 was measured by ELISA (R&D Systems, Minneapolis, MN).

Hematoxylin and Eosin-stained tumor sections were graded by two veterinary pathologists with 100% consensus according to standard criteria.^30^

Sections of the normal, lesion-free, mucosa were formalin-fixed, paraffin-embedded, sectioned, transferred to slides and then deparaffinized. Slides were then probed with primary antibodies for Ki-67 (D3B5, #12202), cleaved caspase-3 (#9661) and CD45 (D3F8Q, #98819) and Tom20 (DBT4N, #42406) (all from Cell Signaling Technologies, Danvers MA) and pancytokeratin (#ab9377. Abcam, Cambridge, MA) on a Ventana Discovery Ultra (Roche, Basel, Switzerland) automated stainer. Detection was achieved using a HRP conjugated goat anti-rabbit secondary antibody (Vector labs, Newark, California), and tyramide signaling amplification (TSA) based detection using Opal fluorophores (Akoya Biosciences, Marlborough, MA). Finally, slides were counter stained with DAPI (Akoya Biosciences) before cover-slipping.

Fluorescent slides were scanned using a Vectra Polaris Quantitative Pathology Imaging System (Akoya Biosciences). Whole slide scans were segmented into QPTIFFs in Phenochart (v1.1.0, Akoya Biosciences), and regions of interest were selected to undergo unmixing in InForm (v2.6.0, Akoya Biosciences) using spectral libraries specific for endogenous autofluorescence and all the fluorophores used. Unmixed QPTIFFs were then fused as single whole slide image and analyzed using HALO (v3.5.3577, Indica Labs, Albuquerque, NM, USA). The Area Quantification (AQ) module was utilized to determine the total area of immunoreactivity of Tom20 within the annotated mucosa layers. Dye intensity thresholds were adjusted using the real-time tuning field to define the range of positive immunoreactivity, and final analysis output was reported in percentage of total colonic mucosa. The fluorescence HighPlex (HP) module was utilized to determine and quantify cell phenotypes. Individual cell nuclei were first identified and segmented using DAPI stain. Nuclear, cytoplasm and membrane thresholds were adjusted to represent a positive staining of remaining markers in each cell. The algorithm was tested on all images using the real-time tuning field to ensure sensitivity and specificity. By applying appropriate inclusion and exclusion criteria, non-leukocyte proliferating cells (CD45^−^, Ki67^+^), and apoptotic cells (cleaved caspase-3^+^) were quantified, and the final analysis output was reported as the percentage of each cell phenotype standardized to the total number of cells in the annotated colonic mucosa (% positive cells).

Total RNA was isolated colon mucosal scraping using Trizol (Thermo Fisher) and then quantified and quality checked using a Fragment Analyzer (Agilent, Santa Clara, CA). One hundred and twenty-five ng of RNA was then used as input for library preparation using a stranded mRNA Library preparation kit following manufacturer instructions (Illumina, San Diego, CA). The resulting libraries were then quantified, mixed in equi-molar concentrations, and sequenced on an Illumina NovaSeq 6000 using a S-Prime 100-cycle kit using single read 100 bases format. Base calling and demultiplex were then performed on an Illumina DRAGEN server using bclconversion. Raw RNA-seq reads (FASTQ) were aligned to the mouse genome (mm10, Genome Reference Consortium Mouse Build 38) using STAR 2.7.10a^31^ with ’–quantMode GeneCounts’ option to obtain the reads per gene value for each sample. The read count data from each sample were normalized, and statistical tests were performed to determine differentially expressed genes (adjusted *P* value using Benjamini–Hochberg method, <.05) using DESeq2 1.34.0.^31^ Function and pathway analyses were conducted using DAVID Knowledgebase.^31^

Total protein was extracted from colonic mucosa or cell lines with RIPA buffer (Thermo) supplemented with protease inhibitor cocktail (Roche, Indianapolis, IN) and quantified using the BCA Assay (Thermo). Colonic IL-6, IL-1β and Tnf-α abundance were measured by ELISA (R&D systems).

Fecal microbiome sequencing was conducted by Zymo Research Corporation (Irvine, CA). Briefly, fecal DNA was extracted using the ZymoBIOMICS®-96 MagBead DNA Kit (Zymo) and libraries prepared using the 16S NGS Library Prep Kit and proprietary primers targeting V3-V4 of the 16S rRNA gene (Zymo). Cleaned and quantified libraries were sequenced on an Illumina MiSeq instrument with a v3 reagent kit (600 cycles) and 10% PhiX spike-in (Illumina). Unique amplicon sequences variants were inferred from raw reads using the DADA2 pipeline.^32^ Potential sequencing errors and chimeric sequences were also removed with the DADA2 pipeline. Taxonomy assignment was performed using Uclust from Qiime v.1.9.1 with the Zymo Research Database, a 16S database that is internally designed and curated, as reference. Composition visualization, alpha-diversity, and beta-diversity analyses were performed with Qiime v.1.9.1.^33^

Mouse fecal samples were analyzed for eight short chain fatty acids: acetic acid (C2), propionic acid (C3), isobutyric acid (C4), butyric acid (C4), 2-methyl-butyric acid (C5), isovaleric acid (C5), valeric acid (C5) and caproic acid (hexanoic acid, C6) by LC-MS/MS (Metabolon, Morrisville, NC). Briefly, samples were spiked with stable labeled internal standards, homogenized and subjected to protein precipitation with an organic solvent. After centrifugation, an aliquot of the supernatant was derivatized, diluted and injected onto an Agilent 1290 (Santa Clara, CA)/AB Sciex QTrap 5500 (Framingham, MA) LC MS/MS system equipped with a C18 reversed phase UHPLC column. The mass spectrometer was operated in negative mode using electrospray ionization (ESI). The peak area of the individual analyte product ions was measured against the peak area of the product ions of the corresponding internal standards. Quantitation was performed using a weighted linear least squares regression analysis generated from fortified calibration standards prepared immediately prior to each run. LC-MS/MS raw data were collected using AB SCIEX software Analyst 1.6.3 and processed using SCIEX OS-MQ software v1.7.

### Statistical analyses

All data are presented as mean ± SEM. Between group comparisons were made using students T test with correction for multiple comparison by False discovery rate. Tumor incidence was compared by Chi-square test. Significance was accepted when *p* < .05 or Q < 0.1. Correlations between variables assessed with Pearson regression. Data analysis was conducted in GraphPad Prism (v9.4.1, San Diego, CA).

Microbiome comparisons were made with LDA (Linear Discriminant Analysis) Effect Size (Lefse) and Multivariate Association with Linear Models (MaAsLin) tools.^34^ We computed the cosine similarity^35^ between the relative abundance vectors of the microbiomes for each donor-recipient pair, considering all 44 genera we detected in samples, using R Statistical Software (v4.1.2; R Core Team 2021).

## Results

### Old donors display elevated systemic and colonic inflammation

Serum IL-6, as well as colonic IL-6, Tnf-α and IL-1β were all significantly higher in DO compared to DY mice. In contrast, there was no apparent difference in gut permeability, assessed by serum FD4 and LPS, between DY and DO mice (Table 1).

**Table 1.** Donor serum and colonic analytes.

	DY	DO	P
Serum FD4 (µg/ml)	5.4 ± 0.2	5.49 ± 0.4	0.97
Serum LPS (EU/ml)	22.2 ± 5.3	13.0 ± 2.5	0.13
Serum IL-6 (pg/ml)	77.6 ± 1.28	116.2 ± 9.78	0.001
Colonic IL-6 (pg/mg protein)	77.6 ± 2.5	102 ± 5.0	0.0004
Colonic IL-1β (pg/mg protein)	132.4 ± 3.0	162.3 ± 4.4	<0.0001
Colonic TNF-α (pg/mg protein)	142.2 ± 8.9	184.6 ± 7.4	0.002

N= 8–10/group.

### Recipient body weight and gut permeability not affected by donor age

**Figure 2.**
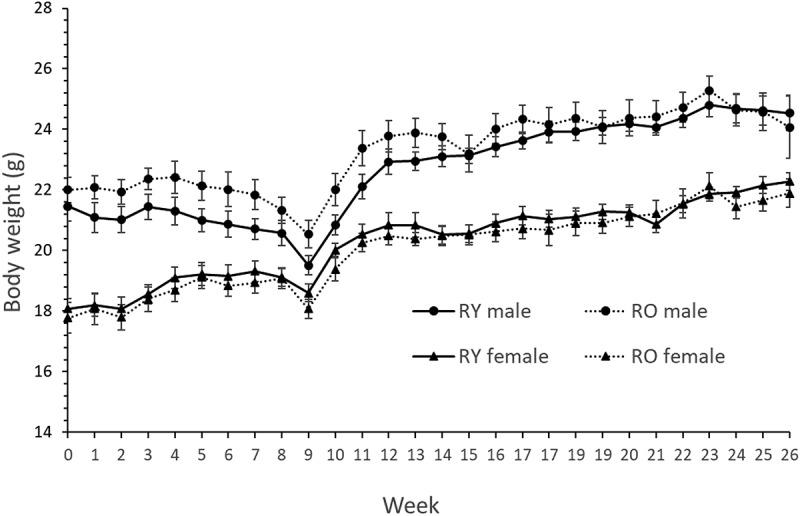
Body weight of recipient mice.

RY, Recipients of FMT from Young donors; RO, Recipients of FMT from Old donors. *N* = 9–10/gp/sex. Repeated measures ANOVA: Males *P* = .22, Females *P* = .8.

Three recipient mice died unexpectedly during the study: one during antibiotic treatment, one during AOM treatment and one during MRI for body composition. Recipient body weight declined an average of 6% from previous peak values because of AOM treatment but recovered rapidly after cessation (Figure 2). Donor age did not affect recipient body weight or composition (Figure 2, Table 2). Serum LPS binding protein (LPB) did not differ between recipients of young or old FMT (2.4 ± 0.1 v 2.4 ± 0.18 µg/ml. *P* = 0.7).

**Table 2.** Body weight and composition of recipient mice.

	Sex	RY	RO	P
Body Weight (g)	M	24.5 ± 0.6	24.1 ± 1.02	0.7
	F	22.3 ± 0.3	21.9 ± 0.5	0.5
Lean Mass (g)	M	19.4 0.2	19.6 0.4	0.7
	F	17.0 ± 0.1	16.8 ± 0.2	0.4
Fat Mass (g)	M	4.0 ± 0.2	3.7 ± 0.3	0.5
	F	3.6 ± 0.2	3.4 ± 0.2	0.5

M, male; F, Female. *N*=18–19/group.

### Recipients of old donors display higher incidence of colonic tumors, higher colonic proliferation and inflammatory cytokines compared to recipients of young donors

**Figure 3.**
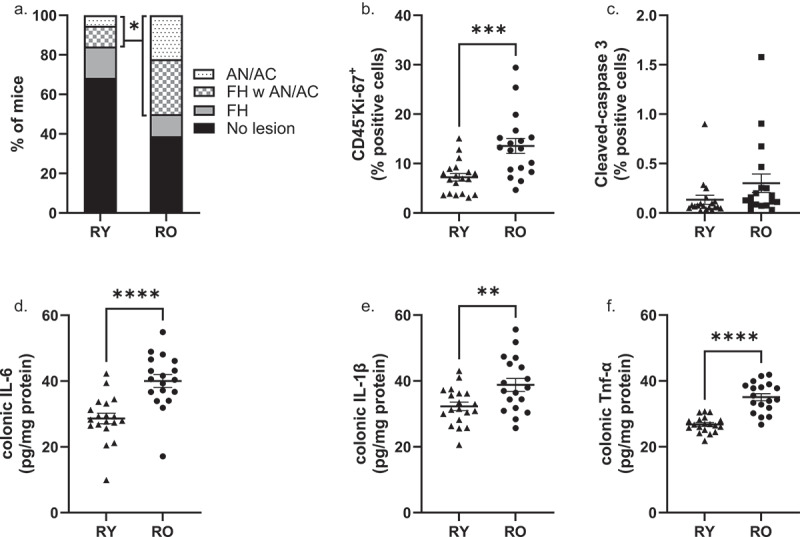
Colonic lesions, cell kinetics and cytokine abundance in recipient mice.

Colonic lesions were graded as focal hyperplasia (FH), adenoma (AN) or adenocarcinoma (AC). In a few instances, lesions were classified as indeterminant adenoma or adenocarcinoma because the submucosa was lost during processing. AN and AC were pooled together for analysis. Tumor data are presented in Figure 3a. Amongst RY mice, 13 (68%) had no detectable lesions, 3 (15.8%) had FH, 2 (10.5%) had both FH and AN/AC, and 1 (5.3%) had only AN/AC. Amongst RO mice, 7 (39%) had no detectable lesions, 2 (11.1%) had FH, 5 (27.8%) had both FH and AN/AC and 4 (22.2%) had only AN/AC. The incidence of AN/AC (with or without FH) was 15.8% in RY and 50% in RO (Chi_2_ = 0.03. RR = 1.7 [1.065–2.970]). The incidence of any lesion (AH and/or AN/AC) was 31.6% and 61.1% in RY and RO, respectively (Chi_2_ = 0.07).

(a) Frequency of mice with colonic lesions. FH, focal hyperplasia; AN/AC, adenoma or adenocarcinoma; *15.8% and 50% of RY and RO mice presented with AN/AC, respectively. Chi_2_ P=.03. (b) percentage of colonocytes that are proliferative from tissue sections, *** P=.0005. (c) percentage of colonocytes that are apoptotic cells from tissue sections. (d) colonic IL-6 concentrations, (e) colonic IL-1β concentrations, (f) colonic Tnf-α concentrations. Data = mean ± SEM (B-F). ****P < .0001. **P = .01. RY, FMT recipients from young donors (N = 19); R O, FMT recipients from old donors (N = 18).

Colon tissue kinetics were evaluated with IHC. The average abundance of proliferating non-leukocytes cells (CD45^−^, Ki-67^+^) in RO colonic sections was approximately double that observed in RY mice (Figure 3b. *P* = .005). In contrast, the abundance of apoptotic cells did not differ between RY and RO mice (Figure 3c). Colonic IL-6, IL-1β and Tnf-α protein concentrations were all significantly higher, by 20–40%, in RO compared to RY mice (Figure 3d-f. *P* < .05).

### Donor age affects colonic transcriptome of recipients with striking enrichment of mitochondria-related genes amongst recipients of old donors

The colon mucosal transcriptome of recipient mice was profiled by RNA sequencing. An average of 7.6 × 10_6_ reads were retrieved per sample (range 3.1 × 10_6_ − 1.4 × 10_7_), and a total of 36 samples were successfully sequenced (*N* = 19, 17 for RY, RO). DeSeq2 analysis identified 2004 genes for which expression was significantly higher in RY mice and 2077 genes in RO mice (Figure S1A). Principal Component Analysis (PCA) revealed a global separation in the gene expression of RY and RO mice (Figure S1B). To determine the functional consequences of genes showing differential expression, we examined their association with established pathways from the Kyoto Encyclopedia of Genes and Genomes (KEGG;^36^), as well as three sub-ontologies from the Gene Ontology knowledgebase^37^: metabolic functions, cellular compartments, and biological processes (Table S1). Of note, amongst the genes more highly expressed in RO mice, there was a striking enrichment of genes related to mitochondrial (Mt) function. Specifically, of the top 5 KEGG pathways identified, 3 related to Mt function and of the top 10 cellular compartments associated with the genes, 5 were assigned to mitochondria. Furthermore, of the top 10 biological processes, half are associated with mitochondrial respiration. Moreover, 41 genes across all 5 oxidative complexes of the Oxidative Phosphorylation pathway were expressed significantly higher in RO compared to RY mice (Figure S2A). In contrast, genes more highly expressed in RY mice tended to be associated with a cytoplasmic or nuclear localization and were related to the processes of cell division, DNA repair and response to DNA damage (Table S1). The apparent unidirectional change of Mt-related genes amongst RO mice is suggestive of increased Mt biogenesis. We therefore stained for Mt in colon sections. In the colonic mucosa, expression of the mitochondrial marker TOM20 was primary in the mucosal epithelial cells confirmed by co-staining of pancytokeratin. We observed an ~34% greater amount of positive staining of TOM20 in the colonic mucosa than in RO compared to RY mice (*P* = 0.006. Fig S2B). Two transcription factors that are known to elicit Mt biogenesis, Esrra and Ppargc1b, were also found to be higher expressed in RO compared to RY colons (Figure S2A).

Overall, our pathway analyses did not detect significant alterations of pathways related to inflammation, such as JAK/STAT (mmu04630) or NF-β (mmu04064), in either group. However, manual review of the latter pathway identified key members that were upregulated in RO mice including Myd88 (20% higher q = 0.005), RelA (p65. 25% higher, q = 0.006) and RelB (42% higher, q = 0.001).

### Old mice display less fecal microbial diversity than young mice

**Figure 4.**
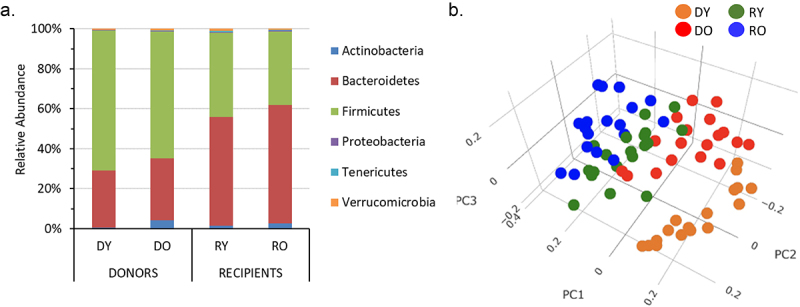
Fecal microbial profiles of donor and recipient mice.

16S sequencing data was obtained for 72 samples with an average read depth of 35,704 ± 1,230 reads per sample (range 12,304–54,064). These samples include 35 matched donor-recipient pairs. The vast majority (~94–98%) of taxa detected belonged to *Bacteroidetes* and *Firmicutes* phyla (Figure 4a). The ratio of *Bacteroidetes:Firmicutes* averaged 2.5 ± 0.3 in donors and 0.8 ± 0.4 in recipients and did not differ between by age within the groups (*P* > .05. Not shown). Microbial alpha diversity measures Chao1 index and Observed species, but not Shannon index PD whole tree or Simpson measures, were significantly lower in old compared to young donors (Table 3). In contrast, none of the alpha diversity measures differed amongst recipients by donor age (Table 3). Beta diversity analyses, assessed by Bray-Curtis dissimilarities, revealed that each specific group had relatively distinct community structures (Figure 4b).

(a) Relative abundance at phylum level. (b) 3-dimensional principal coordinate analysis (PCoA) plot created using the matrix of paired-wise distance between samples calculated by the Bray-Curtis dissimilarity using unique amplicon sequence variants (ASV). *N* = 17 - 19/gp. Males and females pooled together.

**Table 3.** Fecal microbial alpha diversity measures of donor and recipient mice.

	Donors			Recipients		
	DY	DO	P	RY	RO	P
Chao 1 index	178.6 ± 4.5	159.3 ± 5.7	0.01	125.2 ± 6.0	124.3 ± 6.4	0.9
Shannon index	5.45 ± 0.07	5.5 ± 0.1	0.73	4.7 ± 0.1	4.6 ± 0.1	0.5
Observed Species	169.6 ± 3.8	154.6 ± 5.1	0.02	120.7 ± 5.6	118.3 ± 6.1	0.8
PD whole tree	8.2 ± 0.3	8.4 ± 0.2	0.6	7.5 ± 0.2	7.9 ± 0.2	0.2
Simpson	0.94 ± 0.0	0.95 ± 0.01	0.6	0.91 ± 0.01	0.91 ± 0.01	0.96

N=17–19/gp.

### FMT resulted in good engraftment for most genera

To evaluate the effectiveness of microbiota engraftment, we computed the cosine similarity between each donor-recipient pair. Remarkably, all pairs exhibited very high similarity (mean = 0.83 ± 0.02 [max possible = 1.0]) in terms of microbiome composition – even though fecal material was collected from recipients approximately 6 months after the first FMT (Figure S3). Furthermore, we scrutinized the presence of individual genera across all samples. Ten genera were present in all 35 donors, and these were also detected in an average of 32 (91%) or recipients (Table S2). Together, these taxa represent 93% and 91% of fecal bacterial abundance in donors and recipients, respectively. Additionally, we identified 18 taxa that were transferred from donors to recipients 50% of the time or less. However, the combined abundance of these taxa was very low, accounting for only 7% of the total fecal bacteria in donors and 2% in recipients. Thus, overall, we conclude that there was a successful transfer of most of the microbiota community from donors to recipients that persisted throughout the study.

### Specific taxa differ in abundance due to age, donor age and presence of colonic tumors

**Figure 5.**
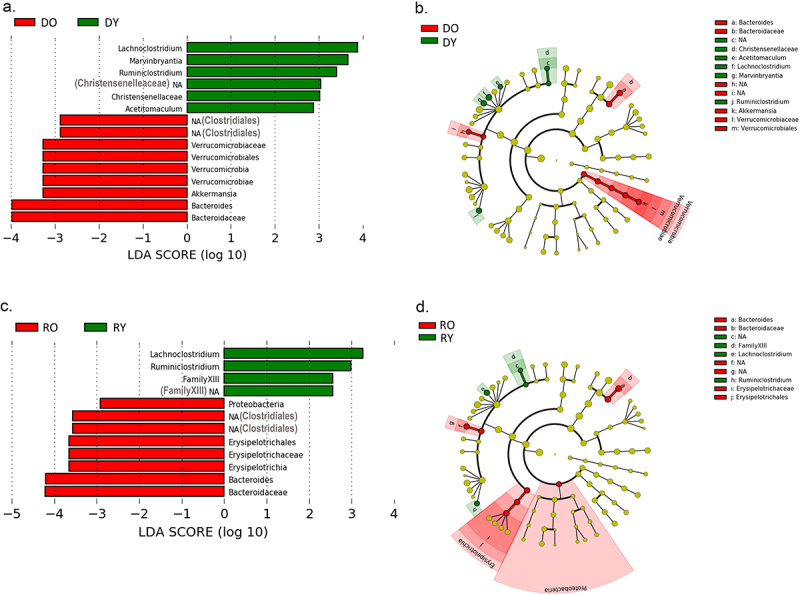
LDA effect size analysis for in donor and recipient mice.

*Lefse* analysis was conducted at a genus level to identify discriminatory taxa according to donor age in donors and recipients. Amongst donor mice, *Lachnoclostridium* and *Marvinbryantia* (both of family *Lachnospiraceae*) were the most discriminatory genera for DY mice, while *Akkermansia* and *Bacteroides* were the most discriminatory for the DO group (Figure 5a,b). Amongst recipient mice, *Lachnoclostridum* and *Rumiosclostridium* were most strongly discriminatory for RY mice while *Bacteroides*, *Erysipelotrichaceae* and an unidentified genus of the order *Clostridiales* were most discriminatory for RO mice (Figure 5c,d).

Lefse outputs showing taxa significantly discriminatory for DY and DO mice (a) and RY and RO mice (c). Cladograms showing the phylogenetic location of taxa differing between DY and DO mice (b) and RY and RO mice (d). Colored nodes indicate the specific taxa that are significantly different. DY, Young Donor; DO, Old Donor. RY, Recipient of Young; RO, Recipient of Old. *N* = 17–19/gp. Sex used as a cofactor in analysis.

**Figure 6.**
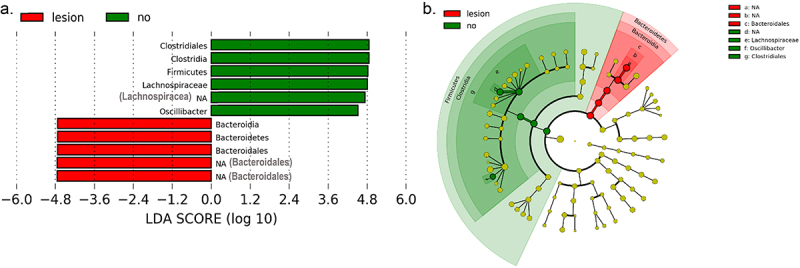
Differences in fecal microbiome between recipient mice with and without colonic lesions.

Microbiomes were further compared between recipient mice based on the presence of any lesion (FH, AN or AC) irrespective of FMT donor age (*N* = 17 with lesions and 20 without). *Lefse* analysis identified members of the order *Clostridiales* (family *Lachnospiraceae* and genus *Oscillibacter*) as being most discriminatory for mice without tumors. Unclassified members of the *Bacteroidales* order were most discriminatory for tumor bearing mice (Figure 6a,b).

(a) Lefse output showing taxa significantly discriminatory for mice with and without colonic lesions (AH, AN or AC). (b) Cladograms showing the phylogenetic location of taxa differing between recipients with and without colonic lesions. Colored nodes indicate the specific taxa that are significantly different. *N* = 17 and 20 mice with and without lesions respectively. Sex used as a cofactor in analysis.

### Fecal short chain fatty acids differ little between groups

Fecal SCFA concentrations are reported in Table 4. In general, fecal SCFA concentrations differed little amongst donors, except for propionic acid, which was ~33% higher in DO compared to DY mice (*P* = .04). Amongst recipient mice, butyric acid was ~27% lower (*P* = .05) in feces of RO compared to RY mice. Further, SCFA concentrations did not differ between mice without colonic lesions, with any lesion and with tumors (366.0 ± 41.1 vs. 277.6 ± 22.9 (*p* = .08) and 288.9 ± 27.0 (*P* = .2) respectively).

**Table 4.** Fecal SCFA content of donor and recipient mice.

	Donors			Recipients		
	DY	DO	P	RY	RO	P
2-Methylbutyric acid (µg/g)	8.9 ± 1.1	9.4 ± 1.7	0.81	5.3 ± 0.5	5.3 ± 0.6	0.92
Acetic acid (µg/g)	663.3 ± 61.2	877.5 ± 83.9	0.04	813.0 ± 71.1	794.3 ± 63.9	0.84
Butyric acid (µg/g)	352.5 ± 45.2	413.8 ± 40.4	0.32	436.4 ± 72.8	274.5 ± 26.3	0.04
Hexanoic acid (µg/g)	1.0 ± 0.1	1.1 ± 0.1	0.70	0.8 ± 0.1	0.8 ± 0.1	0.92
Isobutyric acid (µg/g)	13.1 ± 1.5	13.2 ± 1.8	0.97	12.3 ± 0.9	10.8 ± 0.7	0.24
Isovaleric acid (µg/g)	13.1 ± 1.9	12.7 ± 1.8	0.90	8.1 ± 0.8	6.3 ± 0.8	0.22
Propionic acid (µg/g)	108 ± 10.9	138.3 ± 12.8	0.08	171.6 ± 10.7	150 ± 7.5	0.11
Valeric acid (µg/g)	18.8 ± 3	22.2 ± 2.5	0.40	12.4 ± 1.2	10.7 ± 1.3	0.37

N=18–19 per group. None of the comparisons remained significant after correction for multiple comparisons (Q<0.1).

Relationships between fecal SCFA content and microbes were assessed by Pearson correlation (Figure S4). Interestingly, different patterns were observed in donors and recipients. In donors, the genera most strongly related to SCFA were *Oscillibacter-Oscillospira* and *Erysipelatoclostridium* while in recipients the genera most strongly related to SCFA were *Clostridium, Tyzzerella*, an unclassified genus of family *Ruminococcaceae* and an unclassified genus of class *Mollicutes*. Differences in these patterns likely reflect the differences in the composition of microbiota between donors and recipients and as well as their own genetic differences (C57BL6/J donors, A/J recipients).

## Discussion

In the current study, we observed that mice receiving FMT from old donor mice have an approximately three-fold higher prevalence of colonic tumors (adenoma or adenocarcinoma) compared to mice receiving FMT from young donors. In addition, RO mice displayed an approximately two-fold higher abundance of proliferative cells in their normal colonic epithelium. Others have previously shown that fecal transplant from old to young mice promotes expression of inflammatory mediators in the colon,^23^ cognitive decline^24–27^ retinal degradation and increased microglial activation in the brain,^38^ and metabolic disturbances,^28^ however this is the first indication that microbial communities from older organisms are more carcinogenic than those from younger organisms.

Multiple differences were detected between the fecal microbiome of young and old donor mice. In addition to a modest reduction in alpha diversity, *Lefse* analysis revealed that the genera *Bacteroides, Akkermansia* and an uncharacterized genus of the order *Clostridiales* to be elevated in DO mice. The genera *Lachnoclostridium, Marvinbryantia*, an uncharacterized genus of family *Christensenellaceae* and *Ruminoclostridium* were higher in DY mice.

Amongst recipients, donor age had no discernable effect on alpha diversity. Genera elevated in RY included *Ruminoclostridium, Lachnoclostridium* and an uncharacterized genus of *Clostridiales familyXIII*. Genera elevated in RO included *Bacteroides, Erysipelotrichaceae* and an uncharacterized genus of the order *Clostridiales*. Four genera differed significantly by age in both donors and recipients: *Ruminoclostridium, Lachnoclostridium, Bacteroides* and the uncharacterized genus of the order *Clostridiales* (and in the same direction). These age-related changes are mostly consistent with data from human studies which show that families *Lachnospiraceae* and *Ruminococcaceae* (to which the genera *Lachnoclostridium* and *Ruminoclostridium* belong respectively) decrease, while the genus *Akkermansia* increases, with increasing age. In contrast, while we see an enrichment of *Bacteroides/Bacteroideacea* in DO and RO mice, these taxa are reported to be reduced with advancing age in humans.^39,40^

Based on our cosine similarity analysis (Figure S3), as well as comparison of the presence of individual genera between donors and recipient pairs (Table S2), we conclude that the success of FMT engraftment in this study was relatively high – especially for higher abundance genera. An important consideration for microbiota transplant studies pertains to the choice of recipient mice. Previous work in the aging microbiome field has utilized both germ free (GF)^23,27,28^ and antibiotic-treated recipients.^24–26,38^ Here, we chose the latter approach to enable the use of AOM-sensitive A/J mice. The use of GF recipients may further enhance engraftment of donor microbes, particularly amongst the lower abundance taxa, and confirming our findings using this approach is warranted.

Amongst the most successfully transferred bacteria was an unclassified genera of family *Lachnospiraceae* which accounted for an average of 21% of all fecal microbes in recipient mice. Interestingly, this taxon was less abundant in recipient mice with colonic lesions (FH and/or AN/AC) compared to those without (Figure 6). This observation fits well with data from multiple human studies which show that *Lachnospiraceae* is relatively depleted in CRC patients relative to healthy controls.^5,9,41,42^ Based on these findings, it is tempting to speculate that the lower abundance of the *Lachnospiraceae* genera could contribute to the development of a pro-tumorigenic milieu in the colon. *Lachnospiraceae* have the capacity to produce SCFA;^43^ although we observed a strong relationship between the *Lachnospiraceae* genus *Tyzzerella* and fecal butyrate and hexanoic acid concentrations, the unclassified genera depleted in tumor bearing mice was not related to any SCFA (Figure S3). Thus, if depletion of these genera is indeed mechanistically related to the production of a pro-tumorigenic milieu, our data do not support a role for fecal SCFA as mediators. One alternate potential mechanism linking microbiota shifts to increased risk of colon tumorigenesis involves differential production of secondary bile acids which can affect proliferation and DNA damage.^44^

Interestingly, we observed a moderately lower fecal butyrate (27%) in RO compared to RY mice (*P* = 0.05). There is a considerable body literature demonstrating a tumor suppressive effect of butyrate in the colon (reviewed in).^45,46^ For example, mice colonized with butyrate producing bacteria and fed a high fiber diet or fed a butyrate fortified diet were protected against AOM/DSS induced colon tumorigenesis compared to mice without butyrate producing bacteria regardless of their fiber intake.^47^ Because fecal butyrate did not significantly differ between mice with and without colonic lesions or tumors, it does not appear to be major driver of differences in tumor incidence between RY and RO mice seen here. Furthermore, none of the other SCFAs differed between recipient mice with and without colonic lesions.

Another high abundance taxon that was successfully engrafted in recipients was an unclassified genus with the order *Bacteroidales* (Table S2). This genus was also enriched amongst tumor-bearing mice (Figure 6), a finding that fits well with human observational studies^48^ and with human to mouse FMT studies^11^ associating members of this order with higher tumor burden.

Because others have reported that microbiota from older mice is proinflammatory,^23^ we measured the abundance of pro-inflammatory cytokines in the colon. In agreement with previous reports, we noted that colonic IL-6, IL-1β and Tnf-α concentrations were ~40% (*P* < .0001), ~20% (*P* = .01) and ~31% (*P* < .0001) higher, respectively, in RO than RY mice, thus confirming a more pro-inflammatory capacity of microbial communities originating from old compared to young mice. Importantly, these inflammatory markers were also elevated in old vs young donor mice. Inflammation is a well-accepted risk factor for CRC affects most of the major processes that govern the evolution of a tumor including mutation, proliferation, apoptosis, vascularization, invasion and immune evasion.^17^ Here, colonic IL-6 and Tnf-α were positively correlated to the percentage of proliferative colonocytes (*R* = 0.37, *P* = .02 and *R* = 0.45, *P* = .005 respectively).

To gain further mechanistic insights as to how the molecular milieu of the colon might predisposed to increased tumor formation in RO mice relative to RY mice, we subsequently profiled the colonic transcriptome by RNA-seq. Overall, we saw a striking upregulation of mitochondria-related genes in the RO mice, including components of all five Oxidative Phosphorylation complexes. Because the Mt genes all changed in the same direction, and at a similar magnitude (average ~35% higher in RO), we questioned whether the difference could be an indication of increased Mt biogenesis in RO compared to RY mice. IHC staining for the Mt marker Tom20 confirmed an ~34% greater Mt abundance in colonic mucosa of RO compared to RY mice (*P* = 0.006). As discussed above, RO mice also displayed approximately two-fold more proliferative cells in their normal colonic epithelium than RY mice. Although it is unclear whether increased Mt abundance is a cause or effect of the increased proliferation seen here, it is thought that increased Mt abundance can promote tumorigenesis by providing increased energy for proliferation and/or increased ROS production which may subsequently damage DNA and other macromolecules.^49^

Mitochondrial biogenesis can be induced by the transcription factor peroxisome proliferator-activated receptor-γ coactivator 1β (PGC-1β/Ppargc1b) in concert with Nuclear Respiratory Factor 1 (Nrf1) and Estrogen Related Receptor Alpha (Esrra).^49,50^ Here, we observed significantly higher expression of Ppargc1b (37% higher) and Esrra (53% higher) in RO compared to RY mice – thus constituting a plausible signal to initiate the observed Mt biogenesis. Importantly, Ppargc1b and Esrra have both been implicated in CRC.^51–53^ The impetus for the apparent increased Mt biogenesis amongst RO mice seen here is unclear. Despite being metabolized in the mitochondria, our data do not support a role for lower butyrate in triggering this program because there was no correlation between fecal butyrate (or any other SCFA) and colonic Tom20 staining (*P* > .05) and furthermore butyrate is generally considered to be a promoter of biogenesis.^54^

Several limitations of this study are evident. Firstly, the inclusion of an autologous transplant recipient group would have allowed us to better understand whether the old FMT increased tumorigenesis or the young FMT decreased tumorigenesis, or both. Secondly, it is thought that the second step in the conversion of AOM to the active carcinogen methyldiazonium is conducted by gut microbes.^55,56^ Because we challenged mice with AOM after FMT, we cannot exclude the possibly that the different microbial communities originating from DY and DO mice have a different capacity to metabolize AOM – although we consider this possibility unlikely or at least to have a very minor contribution. Future studies using genetic models of CRC or in which AOM is given before FMT could exclude this possibility. Thirdly, we utilized C57BL6J donor mice because older ages are readily commercially available. A potential artifact arises from our use of A/J mice, rather than C57BL6J mice, as recipients because the latter are relatively insensitive to AOM-induced colorectal tumorigenesis.^57^ This strain mismatch between donor and recipients may limit the extent of engraftment of microbes from donors to recipients. Finally, the inability of 16S sequencing to provide specifies-level taxa identifications (and higher taxonomic levels in many cases) precludes us from identifying specific species that could be administered alone or in combinations in efforts to recreate the differences in tumor rate.

Despite these limitations, this study provides the first proof-of-concept that age-altered gut microbial communities *per se* are more tumorigenic, and promote higher colonic inflammation and proliferation, than communities originating from young animals. Although a snapshot in time after the period in which tumors were forming, analyses of normal colonic epithelium of recipient mice (collected at the end of the study) unearthed a plausible mechanism for higher colonic tumor formation in RO than RY mice. Specifically, Ppargc1b/Esrra mediated upregulation of mitochondrial biogenesis could fuel increased colonic proliferation while leaking DNA damaging ROS. Elevated levels of colonic inflammation may further encourage tumorigenesis in the colon of RO mice. Several differences were identified between the microbial profile of DY and DO mice as well as between RY and RO mice, however it remains unclear which specific microbes are responsible and how, mechanistically, they might incite a more pro-tumorigenic program in the colonic epithelium of RO mice. Somewhat surprisingly, the current data do not support a prominent role for SCFA as the mediator here. More work is needed to confirm these findings using additional animal models and human donor samples, and to better elucidate the mechanisms involved. These data raise the possibility that microbial manipulations, such as FMT from young to old, could be used to attenuate the increased risk for CRC seen with increasing age.

## Supplementary Material

Table S1. Pathway analysis.docx

Figure S1. RNA seq summary.docx

Figure S2. Mitochondrial genes and staining.docx

Figure S4. SCFA vs taxa heatmap.docx

Table S2. Engraftment analysis revised.docx

Figure S3. FMT transplant efficiency.docx

## Data Availability

RNA-Seq and 16S rRNA sequencing data are deposited at the NIH Gene Expression Omnibus (GEO) under accession #GSE237337.

## References

[1] Siegel RL, Miller KD, Jemal A. Cancer statistics, 2016. CA Cancer J Clin. 2016;66(1):7–16. doi:10.3322/caac.21332. 26742998

[2] Bowel cancer incidence statistics cancer Research UK

[3] U.S. Cancer statistics working group. U.S. Cancer statistics data visualizations tool, based on 2021 submission data (1999-2019). U.S. Department of Health and Human Services, Centers for Disease Control and Prevention and National Cancer Institute. https://www.cdc.gov/cancer/dataviz

[4] An aging nation United States Census Bureu

[5] Wang T, Cai G, Qiu Y, Fei N, Zhang M, Pang X, Jia W, Cai S, Zhao L. Structural segregation of gut microbiota between colorectal cancer patients and healthy volunteers. ISME J. 2012;6(2):320–329. doi:10.1038/ismej.2011.109. 21850056 PMC3260502

[6] Ahn J, Sinha R, Pei Z, Dominianni C, Wu J, Shi J, Goedert JJ, Hayes RB, Yang L. Human gut microbiome and risk for colorectal cancer. J Natl Cancer Inst. 2013;105(24):1907–1911. doi:10.1093/jnci/djt300. 24316595 PMC3866154

[7] McCoy AN, Araujo-Perez F, Azcarate-Peril A, Yeh JJ, Sandler RS, Keku TO, Goel A. Fusobacterium is associated with colorectal adenomas. PLoS ONE. 2013;8(1):e53653. doi:10.1371/journal.pone.0053653. 23335968 PMC3546075

[8] Shen XJ, Rawls JF, Randall T, Burcal L, Mpande CN, Jenkins N, Jovov B, Abdo Z, Sandler RS, Keku TO. Molecular characterization of mucosal adherent bacteria and associations with colorectal adenomas. Gut Microbes. 2010;1(3):138–147. doi:10.4161/gmic.1.3.12360. 20740058 PMC2927011

[9] Peters BA, Dominianni C, Shapiro JA, Church TR, Wu J, Miller G, Yuen E, Freiman H, Lustbader I, Salik J, et al. The gut microbiota in conventional and serrated precursors of colorectal cancer. Microbiome. 2016;4(1):69. doi:10.1186/s40168-016-0218-6. 28038683 PMC5203720

[10] Hale VL, Chen J, Johnson S, Harrington SC, Yab TC, Smyrk TC, Nelson H, Boardman LA, Druliner BR, Levin TR, et al. Shifts in the fecal microbiota associated with adenomatous polyps. Cancer Epidemiol Biomarkers Prev. 2017;26(1):85–94. doi:10.1158/1055-9965.EPI-16-0337. 27672054 PMC5225053

[11] Baxter NT, Zackular JP, Chen GY, Schloss PD. Structure of the gut microbiome following colonization with human feces determines colonic tumor burden. Microbiome. 2014;2(20): doi:10.1186/2049-2618-2-20. PMC4070349 24967088

[12] Kostic AD, Chun E, Robertson L, Glickman JN, Gallini CA, Michaud M, Clancy TE, Chung DC, Lochhead P, Hold GL, et al. Fusobacterium nucleatum potentiates intestinal tumorigenesis and modulates the tumor-immune microenvironment. Cell Host & Microbe. 2013;14(2):207–215. doi:10.1016/j.chom.2013.07.007. 23954159 PMC3772512

[13] Wu S, Rhee KJ, Albesiano E, Rabizadeh S, Wu X, Yen HR, Huso DL, Brancati FL, Wick E, McAllister F, et al. A human colonic commensal promotes colon tumorigenesis via activation of t helper type 17 t cell responses. Nat Med. 2009;15(9):1016–1022. doi:10.1038/nm.2015. 19701202 PMC3034219

[14] Arthur JC, Perez-Chanona E, Muhlbauer M, Tomkovich S, Uronis JM, Fan TJ, Campbell BJ, Abujamel T, Dogan B, Rogers AB, et al. Intestinal inflammation targets cancer-inducing activity of the microbiota. Sci. 2012;338(6103):120–123. doi:10.1126/science.1224820. PMC3645302 22903521

[15] Zackular JP, Baxter NT, Iverson KD, Sadler WD, Petrosino JF, Chen GY, Schloss PD, Blaser MJ. The gut microbiome modulates colon tumorigenesis. MBio. 2013;4(6):e00692–00613. doi:10.1128/mBio.00692-13. 24194538 PMC3892781

[16] Uronis JM, Muhlbauer M, Herfarth HH, Rubinas TC, Jones GS, Jobin C, Bereswill S. Modulation of the intestinal microbiota alters colitis-associated colorectal cancer susceptibility. PLoS ONE. 2009;4(6):e6026. doi:10.1371/journal.pone.0006026. 19551144 PMC2696084

[17] Karin M. Nuclear factor-kappab in cancer development and progression. Nature. 2006;441(7092):431–436. doi:10.1038/nature04870. 16724054

[18] Franceschi C, Bonafe M, Valensin S, Olivieri F, De Luca M, Ottaviani E, De Benedictis G. Inflamm-aging. An evolutionary perspective on immunosenescence. Ann N Y Acad Sci. 2000;908:908(244–254. doi:10.1111/j.1749-6632.2000.tb06651.x. 10911963

[19] Langille MG, Meehan CJ, Koenig JE, Dhanani AS, Rose RA, Howlett SE, Beiko RG. Microbial shifts in the aging mouse gut. Microbiome. 2014;2(1):50. doi:10.1186/s40168-014-0050-9. 25520805 PMC4269096

[20] Elderman M, Sovran B, Hugenholtz F, Graversen K, Huijskes M, Houtsma E, Belzer C, Boekschoten M, de Vos P, Dekker J, et al. The effect of age on the intestinal mucus thickness, microbiota composition and immunity in relation to sex in mice. PLoS ONE. 2017;12(9):e0184274. doi:10.1371/journal.pone.0184274. 28898292 PMC5595324

[21] Kim KA, Jeong JJ, Yoo SY, Kim DH. Gut microbiota lipopolysaccharide accelerates inflamm-aging in mice. BMC Microbiol. 2016;16(9): doi:10.1186/s12866-016-0625-7. PMC4715324 26772806

[22] Sovran B, Hugenholtz F, Elderman M, Van Beek AA, Graversen K, Huijskes M, Boekschoten MV, Savelkoul HFJ, De Vos P, Dekker J, et al. Age-associated impairment of the mucus barrier function is associated with profound changes in microbiota and immunity. Sci Rep. 2019;9(1):1437. doi:10.1038/s41598-018-35228-3. 30723224 PMC6363726

[23] Fransen F, van Beek AA, Borghuis T, Aidy SE, Hugenholtz F, van der Gaast-de Jongh C, Savelkoul HFJ, De Jonge MI, Boekschoten MV, Smidt H, et al. Aged gut microbiota contributes to systemical inflammaging after transfer to germ-free mice. Front Immunol. 2017;8:1385. doi:10.3389/fimmu.2017.01385. 29163474 PMC5674680

[24] Rei D, Saha S, Haddad M, Rubio AH, Perlaza BL, Berard M, Ungeheuer MN, Sokol H, Lledo PM. Age-associated gut microbiota impair hippocampus-dependent memory in a vagus-dependent manner. JCI Insight. 2022;7(15): doi:10.1172/jci.insight.147700. PMC9462480 35737457

[25] D’Amato A, Di Cesare Mannelli L, Lucarini E, Man AL, Le Gall G, Branca JJV, Ghelardini C, Amedei A, Bertelli E, Regoli M, et al. Faecal microbiota transplant from aged donor mice affects spatial learning and memory via modulating hippocampal synaptic plasticity- and neurotransmission-related proteins in young recipients. Microbiome. 2020;8(1):140. doi:10.1186/s40168-020-00914-w. 33004079 PMC7532115

[26] Li Y, Ning L, Yin Y, Wang R, Zhang Z, Hao L, Wang B, Zhao X, Yang X, Yin L, et al. Age-related shifts in gut microbiota contribute to cognitive decline in aged rats. Aging (Albany NY). 2020;12(9):7801–7817. doi:10.18632/aging.103093. 32357144 PMC7244050

[27] Lee J, Venna VR, Durgan DJ, Shi H, Hudobenko J, Putluri N, Petrosino J, McCullough LD, Bryan RM. Young versus aged microbiota transplants to germ-free mice: increased short-chain fatty acids and improved cognitive performance. Gut Microbes. 2020;12(1):1–14. doi:10.1080/19490976.2020.1814107. PMC7757789 32897773

[28] Binyamin D, Werbner N, Nuriel-Ohayon M, Uzan A, Mor H, Abbas A, Ziv O, Teperino R, Gutman R, Koren O. The aging mouse microbiome has obesogenic characteristics. Genome Med. 2020;12(1):87. doi:10.1186/s13073-020-00784-9. 33046129 PMC7552538

[29] Yu Q, Newsome RC, Beveridge M, Hernandez MC, Gharaibeh RZ, Jobin C, Thomas RM. Intestinal microbiota modulates pancreatic carcinogenesis through intratumoral natural killer cells. Gut Microbes. 2022;14(1):2112881. doi:10.1080/19490976.2022.2112881. 35980869 PMC9397420

[30] Nolte T, Brander-Weber P, Dangler C, Deschl U, Elwell MR, Greaves P, Hailey R, Leach MW, Pandiri AR, Rogers A, et al. Nonproliferative and proliferative lesions of the gastrointestinal tract, pancreas and salivary glands of the rat and mouse. J Toxicol Pathol. 2016;29(1 Suppl):1S–125S. doi:10.1293/tox.29.1S. PMC4765498 26973378

[31] Dobin A, Davis CA, Schlesinger F, Drenkow J, Zaleski C, Jha S, Batut P, Chaisson M, Gingeras TR. Star: Ultrafast universal RNA-seq aligner. Bioinformatics. 2013;29(1):15–21. doi:10.1093/bioinformatics/bts635. 23104886 PMC3530905

[32] Callahan BJ, McMurdie PJ, Rosen MJ, Han AW, Johnson AJ, Holmes SP. Dada2: High-resolution sample inference from illumina amplicon data. Nat Methods. 2016;13(7):581–583. doi:10.1038/nmeth.3869. 27214047 PMC4927377

[33] Caporaso JG, Kuczynski J, Stombaugh J, Bittinger K, Bushman FD, Costello EK, Fierer N, Pena AG, Goodrich JK, Gordon JI, et al. Qiime allows analysis of high-throughput community sequencing data. Nat Methods. 2010;7(5):335–336. doi:10.1038/nmeth.f.303. 20383131 PMC3156573

[34] Segata N, Izard J, Waldron L, Gevers D, Miropolsky L, Garrett WS, Huttenhower C. Metagenomic biomarker discovery and explanation. Genome Biol. 2011;12(6):R60. doi:10.1186/gb-2011-12-6-r60. 21702898 PMC3218848

[35] Zhang C, Cleveland K, Schnoll-Sussman F, McClure B, Bigg M, Thakkar P, Schultz N, Shah MA, Betel D. Identification of low abundance microbiome in clinical samples using whole genome sequencing. Genome Biol. 2015;16(265): doi:10.1186/s13059-015-0821-z. PMC4661937 26614063

[36] Kanehisa M, Furumichi M, Sato Y, Kawashima M, Ishiguro-Watanabe M. Kegg for taxonomy-based analysis of pathways and genomes. Nucleic Acids Res. 2023;51(D1):D587–D592. doi:10.1093/nar/gkac963. 36300620 PMC9825424

[37] Gene Ontology C, Aleksander SA, Balhoff J, Carbon S, Cherry JM, Drabkin HJ, Ebert D, Feuermann M, Gaudet P, Harris NL. The gene ontology knowledgebase in 2023. Genetics. 2023;224(1) .doi:10.1093/genetics/iyad031. PMC10158837 36866529

[38] Parker A, Romano S, Ansorge R, Aboelnour A, Le Gall G, Savva GM, Pontifex MG, Telatin A, Baker D, Jones E, et al. Fecal microbiota transfer between young and aged mice reverses hallmarks of the aging gut, eye, and brain. Microbiome. 2022;10(1):68. doi:10.1186/s40168-022-01243-w. 35501923 PMC9063061

[39] Badal VD, Vaccariello ED, Murray ER, Yu KE, Knight R, Jeste DV, Nguyen TT. The gut microbiome, aging, and longevity: a systematic review. Nutrients. 2020;12(12):3759. doi:10.3390/nu12123759. 33297486 PMC7762384

[40] Biagi E, Franceschi C, Rampelli S, Severgnini M, Ostan R, Turroni S, Consolandi C, Quercia S, Scurti M, Monti D, et al. Gut microbiota and extreme longevity. Curr Biol. 2016;26(11):1480–1485. doi:10.1016/j.cub.2016.04.016. 27185560

[41] Clos-Garcia M, Garcia K, Alonso C, Iruarrizaga-Lejarreta M, D’Amato M, Crespo A, Iglesias A, Cubiella J, Bujanda L, Falcon-Perez JM. Integrative analysis of fecal metagenomics and metabolomics in colorectal cancer. Cancers Basel. 2020;12(5):1142. doi:10.3390/cancers12051142. 32370168 PMC7281174

[42] Zackular JP, Rogers MA, MTt R, Schloss PD. The human gut microbiome as a screening tool for colorectal cancer. Cancer Prev Res (Phila). 2014;7(11):1112–1121. doi:10.1158/1940-6207.CAPR-14-0129. 25104642 PMC4221363

[43] Vacca M, Celano G, Calabrese FM, Portincasa P, Gobbetti M, De Angelis M. The controversial role of human gut lachnospiraceae. Microorganisms. 2020;8(4):573. doi:10.3390/microorganisms8040573. 32326636 PMC7232163

[44] Liu Y, Zhang S, Zhou W, Hu D, Xu H, Ji G. Secondary bile acids and tumorigenesis in colorectal cancer. Front Oncol. 2022;12(813745): doi:10.3389/fonc.2022.813745. PMC9097900 35574393

[45] Fung KY, Cosgrove L, Lockett T, Head R, Topping DL. A review of the potential mechanisms for the lowering of colorectal oncogenesis by butyrate. Br J Nutr. 2012;108(5):820–831. doi:10.1017/S0007114512001948. 22676885

[46] Bultman SJ. Interplay between diet, gut microbiota, epigenetic events, and colorectal cancer. Mol Nutr Food Res. 2016;61. doi:10.1002/mnfr.201500902. PMC5161716 27138454

[47] Donohoe DR, Holley D, Collins LB, Montgomery SA, Whitmore AC, Hillhouse A, Curry KP, Renner SW, Greenwalt A, Ryan EP, et al. A gnotobiotic mouse model demonstrates that dietary fiber protects against colorectal tumorigenesis in a microbiota- and butyrate-dependent manner. Cancer Discov. 2014;4(12):1387–1397. doi:10.1158/2159-8290.CD-14-0501. 25266735 PMC4258155

[48] Hatcher C, Richenberg G, Waterson S, Nguyen LH, Joshi AD, Carreras-Torres R, Moreno V, Chan AT, Gunter M, Lin Y, et al. Application of Mendelian randomization to explore the causal role of the human gut microbiome in colorectal cancer. Sci Rep. 2023;13(1):5968. doi:10.1038/s41598-023-31840-0. 37045850 PMC10097673

[49] Vyas S, Zaganjor E, Haigis MC. Mitochondria and cancer. Cell. 2016;166(3):555–566. doi:10.1016/j.cell.2016.07.002. 27471965 PMC5036969

[50] Shao D, Liu Y, Liu X, Zhu L, Cui Y, Cui A, Qiao A, Kong X, Liu Y, Chen Q, et al. Pgc-1 beta-regulated mitochondrial biogenesis and function in myotubes is mediated by nrf-1 and err alpha. Mitochondrion. 2010;10(5):516–527. doi:10.1016/j.mito.2010.05.012. 20561910

[51] Frodyma DE, Troia TC, Rao C, Svoboda RA, Berg JA, Shinde DD, Thomas VC, Lewis RE, Fisher KW. PGC-1β and ERRα Promote Glutamine Metabolism and Colorectal Cancer Survival via Transcriptional Upregulation of PCK2. Cancers Basel. 2022;14(19):4879. doi:10.3390/cancers14194879. 36230802 PMC9562873

[52] Bellafante E, Morgano A, Salvatore L, Murzilli S, Di Tullio G, D’Orazio A, Latorre D, Villani G, Moschetta A. Pgc-1beta promotes enterocyte lifespan and tumorigenesis in the intestine. Proc Natl Acad Sci U S A. 2014;111(42):E4523–4531. doi:10.1073/pnas.1415279111. 25288742 PMC4210309

[53] Fisher KW, Das B, Kim HS, Clymer BK, Gehring D, Smith DR, Costanzo-Garvey DL, Fernandez MR, Brattain MG, Kelly DL, et al. AMPK promotes aberrant pgc1beta expression to support human colon tumor cell survival. Mol Cell Biol. 2015;35(22):3866–3879. doi:10.1128/MCB.00528-15. 26351140 PMC4609747

[54] Walsh ME, Bhattacharya A, Sataranatarajan K, Qaisar R, Sloane L, Rahman MM, Kinter M, Van Remmen H. The histone deacetylase inhibitor butyrate improves metabolism and reduces muscle atrophy during aging. Aging Cell. 2015;14(6):957–970. doi:10.1111/acel.12387. 26290460 PMC4693467

[55] Reddy BS, Weisburger JH, Narisawa T, Wynder EL. Colon carcinogenesis in germ-free rats with 1,2-dimethylhydrazine and n-methyl-n’-nitro-n-nitrosoguanidine. Cancer Res. 1974;34:2368–2372. 4843537

[56] Papanikolaou A, Wang QS, Delker DA, Rosenberg DW. Azoxymethane-induced colon tumors and aberrant crypt foci in mice of different genetic susceptibility. Cancer Lett. 1998;130(1–2):29–34. doi:10.1016/S0304-3835(98)00101-3. 9751253

[57] Neufert C, Becker C, Neurath MF. An inducible mouse model of colon carcinogenesis for the analysis of sporadic and inflammation-driven tumor progression. Nat Protoc. 2007;2(8):1998–2004. doi:10.1038/nprot.2007.279. 17703211

