# Supplementary material for: Fecal microbiota transplanted from old mice promotes more colonic inflammation, proliferation, and tumor formation in azoxymethane-treated A/J mice than microbiota originating from young mice
Source: Gut Microbes. 2023 Nov 29;15(2):2288187. doi: 10.1080/19490976.2023.2288187 (PMC10730208; doi:10.1080/19490976.2023.2288187)
Supplement: Table S1. Pathway analysis.docx [file KGMI_A_2288187_SM6858.docx]

|  | **Higher in RY (N=1798)** | | | | **Higher in RO (N=1875)** | | | |
| --- | --- | --- | --- | --- | --- | --- | --- | --- |
|  | **Name** | **N genes** | **Fold enrichment** | **FDR** | **Name** | **N genes** | **Fold enrichment** | **FDR** |
| **Cellular Compartment** | GO:0005737~cytoplasm  GO:0005829~cytosol  GO:0005634~nucleus  GO:0005654~nucleoplasm  GO:0005794~Golgi apparatus  GO:0048471~perinuclear region of cytoplasm  GO:0005730~nucleolus  GO:0000139~Golgi membrane  GO:0005694~chromosome  GO:0005783~endoplasmic reticulum | 999  646  857  504  250  159  163  112  114  245 | 1.5  1.7  1.5  1.6  1.9  2.1  2.0  2.2  2.2  1.6 | 1.69E-53  1.47E-45  1.02E-40  2.95E-28  3.65E-22  1.85E-18  4.10E-16  9.62E-14  1.82E-13  4.24E-13 | GO:0005739~mitochondrion  GO:0005743~mitochondrial inner membrane  GO:0005737~cytoplasm  GO:0005829~cytosol  GO:0005840~ribosome  GO:0005654~nucleoplasm  GO:0070469~respiratory chain  GO:0005768~endosome  GO:0005759~mitochondrial matrix  GO:0005747~mitochondrial respiratory chain complex I | 329  140  823  511  46  389  23  109  48  19 | 2.1  3.2  1.3  1.4  2.8  1.3  4.4  1.8  2.3  4.4 | 1.16E-36  4.39E-33  4.66E-21  2.81E-17  5.25E-08  9.24E-08  2.96E-07  7.89E-07  5.55E-06  9.14E-06 |
| **Biological Process** | GO:0007049~cell cycle  GO:0051301~cell division  GO:0006281~DNA repair  GO:0015031~protein transport  GO:0006974~cellular response to DNA damage stimulus  GO:0006511~ubiquitin-dependent protein catabolic process  GO:0016567~protein ubiquitination  GO:0045893~positive regulation of transcription, DNA-templated  GO:0006397~mRNA processing  GO:0016477~cell migration | 128  89  81  113  96  62  86  121  70  63 | 2.1  2.4  2.3  1.9  2.0  2.4  2.1  1.8  2.1  2.2 | 1.74E-12  2.17E-11  1.16E-08  4.22E-08  5.56E-08  1.08E-07  1.12E-07  3.96E-07  5.68E-06  5.68E-06 | GO:0015031~protein transport  GO:0016310~phosphorylation  GO:0009060~aerobic respiration  GO:0042776~mitochondrial ATP synthesis coupled proton transport  GO:0006629~lipid metabolic process  GO:0032543~mitochondrial translation  GO:0007005~mitochondrion organization  GO:0016192~vesicle-mediated transport  GO:0032981~mitochondrial respiratory chain complex I assembly  GO:0006468~protein phosphorylation | 103  101  25  23  102  24  26  48  18  88 | 1.9  1.9  4.2  4.3  1.7  3.0  2.8  2.0  3.4  1.6 | 1.16E-06  1.16E-06  2.20E-06  5.40E-06  4.96E-04  0.003  0.004  0.004  0.006  0.007 |
| **Metabolic Function** | GO:0005515~protein binding  GO:0000166~nucleotide binding  GO:0003723~RNA binding  GO:0016740~transferase activity  GO:0005524~ATP binding  GO:0042802~identical protein binding  GO:0004842~ubiquitin-protein transferase activity  GO:0046872~metal ion binding  GO:0019904~protein domain specific binding  GO:0019901~protein kinase binding | 751  286  165  268  234  278  59  447  72  101 | 1.5  1.8  1.9  1.6  1.6  1.5  2.4  1.3  2.2  1.8 | 1.26E-34  5.50E-20  4.09E-13  7.18E-13  1.37E-11  3.67E-09  4.63E-08  4.96E-08  8.59E-08  2.71E-07 | GO:0000166~nucleotide binding  GO:0016740~transferase activity  GO:0005515~protein binding  GO:0016301~kinase activity  GO:0005524~ATP binding  GO:0003735~structural constituent of ribosome  GO:0016787~hydrolase activity  GO:0042802~identical protein binding  GO:0004674~protein serine/threonine kinase activity  GO:0046872~metal ion binding | 229  232  581  107  202  38  190  227  65  382 | 1.5  1.5  1.2  1.9  1.5  2.3  1.4  1.3  1.7  1.2 | 4.33E-08  9.11E-08  1.84E-07  1.84E-07  3.38E-07  6.41E-04  0.003  0.004  0.004  0.004 |
| **KEGG Pathway** | mmu04144: Endocytosis  mmu04141: Protein processing in endoplasmic reticulum  mmu04140: Autophagy - animal  mmu05205: Proteoglycans in cancer  mmu05132: Salmonella infection  mmu04218: Cellular senescence  mmu05170: Human immunodeficiency virus 1 infection  mmu03013: Nucleocytoplasmic transport  mmu00562: Inositol phosphate metabolism  mmu04120: Ubiquitin mediated proteolysis | 55  39  34  43  49  39  47  28  20  31 | 2.0  2.3  2.4  2.1  1.9  2.1  1.9  2.4  2.8  2.1 | 1.73E-04  2.79E-04  2.79E-04  3.35E-04  4.43E-04  4.43E-04  4.43E-04  9.27E-04  0.002  0.003 | mmu01100: Metabolic pathways  mmu04714: Thermogenesis  mmu04932: Non-alcoholic fatty liver disease  mmu00190: Oxidative phosphorylation  mmu05208: Chemical carcinogenesis - reactive oxygen species  mmu05016: Huntington disease  mmu05012: Parkinson disease  mmu05020: Prion disease  mmu05022: Pathways of neurodegeneration - multiple diseases  mmu05415: Diabetic cardiomyopathy | 233  60  45  41  55  62  55  54  80  46 | 1.6  2.9  3.2  3.4  2.7  2.3  2.3  2.2  1.9  2.4 | 1.18E-12  8.19E-12  2.54E-10  3.26E-10  3.26E-10  4.40E-08  2.26E-07  8.29E-07  8.29E-07  8.29E-07 |

**Table S1. Pathway analysis of genes differentially expressed between recipient groups by RNA seq.**

Pathway analysis conducted in DAVID Knowledgebase.
