# Supplementary material for: Fecal microbiota transplanted from old mice promotes more colonic inflammation, proliferation, and tumor formation in azoxymethane-treated A/J mice than microbiota originating from young mice
Source: Gut Microbes. 2023 Nov 29;15(2):2288187. doi: 10.1080/19490976.2023.2288187 (PMC10730208; doi:10.1080/19490976.2023.2288187)
Supplement: Figure S1. RNA seq summary.docx [file KGMI_A_2288187_SM6857.docx]

**Figure S1. Summary of recipient colon transcriptome analyses**


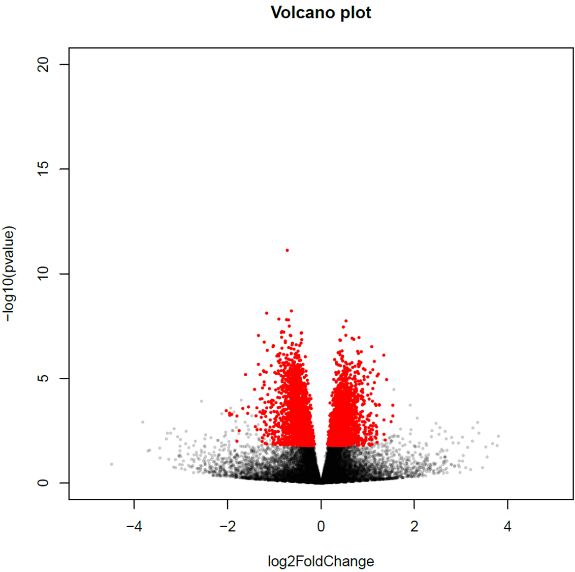


Higher in RO

(n = 2004)

Higher in RY

(n = 2077)


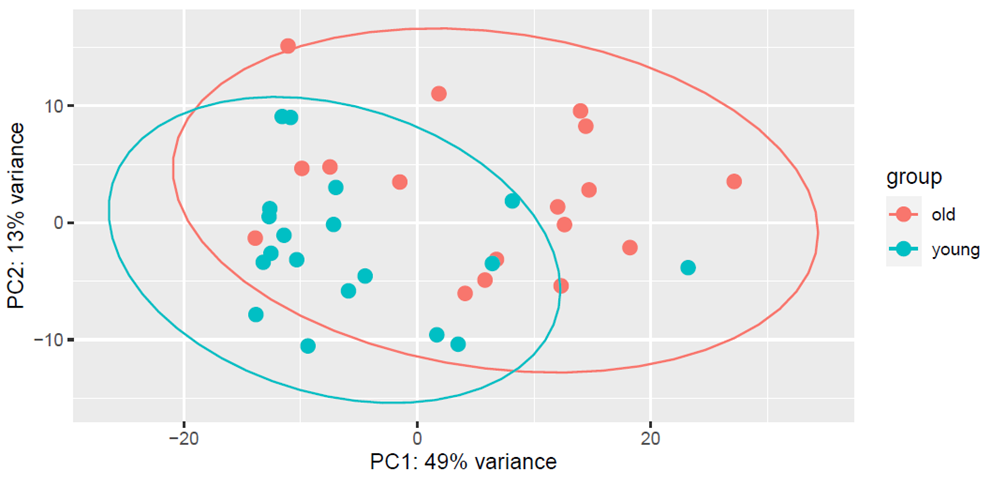


**A.**

**B.**

RO

RY

***A****) Volcano plot showing the number of genes significantly elevated in RY and RO mice. B) Principal Component Analysis. Significant accepted when Benjamini-Hochberg adjusted P<0.05. RY, recipient of Young Donor. RO, Recipient of Old donor. N= 17-19/gp.*
