# Supplementary material for: Fecal microbiota transplanted from old mice promotes more colonic inflammation, proliferation, and tumor formation in azoxymethane-treated A/J mice than microbiota originating from young mice
Source: Gut Microbes. 2023 Nov 29;15(2):2288187. doi: 10.1080/19490976.2023.2288187 (PMC10730208; doi:10.1080/19490976.2023.2288187)
Supplement: Figure S2. Mitochondrial genes and staining.docx [file KGMI_A_2288187_SM6856.docx]

**Figure S2. Mitochondrial gene expression and abundance in recipient colons**


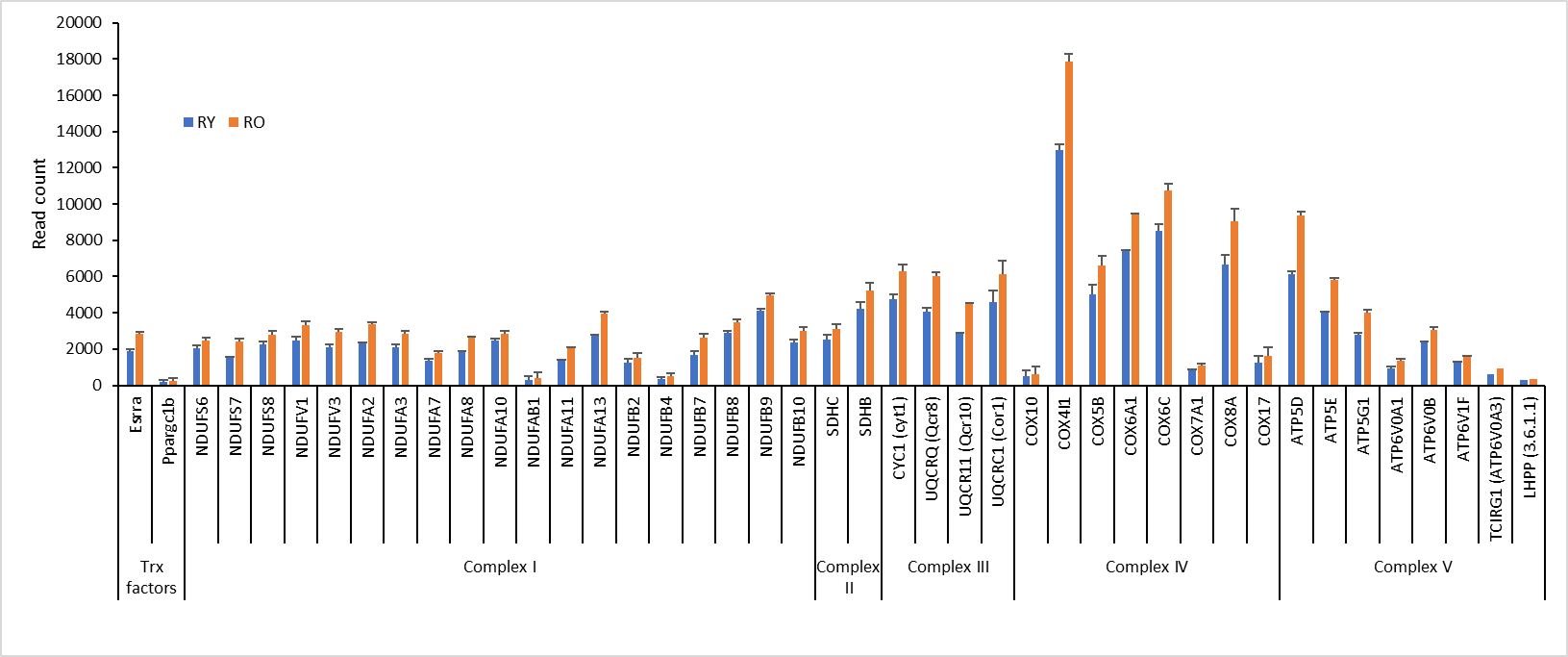

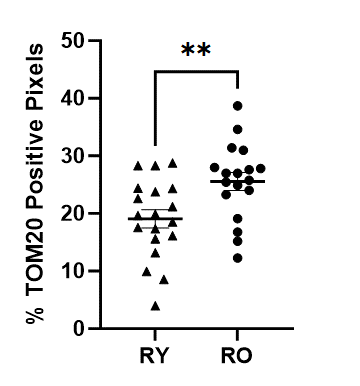


**A)** Mitochondria-related genes significantly upregulated in RO vs RY mice (Padj <0.05). Data = mean ± SEM. **B)** Quantification of TOM20 (mitochondrial marker) staining by fluorescent Immunohistochemistry. **P=0.006. N=18-19/group
