# Supplementary material for: Fecal microbiota transplanted from old mice promotes more colonic inflammation, proliferation, and tumor formation in azoxymethane-treated A/J mice than microbiota originating from young mice
Source: Gut Microbes. 2023 Nov 29;15(2):2288187. doi: 10.1080/19490976.2023.2288187 (PMC10730208; doi:10.1080/19490976.2023.2288187)
Supplement: Figure S4. SCFA vs taxa heatmap.docx [file KGMI_A_2288187_SM6855.docx]

**Figure S4. Associations between fecal SCFA and microbes in donor and recipient mice.**
