# Supplementary material for: Fecal microbiota transplanted from old mice promotes more colonic inflammation, proliferation, and tumor formation in azoxymethane-treated A/J mice than microbiota originating from young mice
Source: Gut Microbes. 2023 Nov 29;15(2):2288187. doi: 10.1080/19490976.2023.2288187 (PMC10730208; doi:10.1080/19490976.2023.2288187)
Supplement: Table S2. Engraftment analysis revised.docx [file KGMI_A_2288187_SM6854.docx]

**Table S2. Engraftment of genera from donor to recipient mice.**

| GENUS | Average abundance | | Donors Positive | | Donors, Recipients both positive | |
| --- | --- | --- | --- | --- | --- | --- |
|  | Donors | Recipients | N | % | N | % |
| p__Bacteroidetes;c__Bacteroidia;o__Bacteroidales;f__NA;g__NA | 0.2890796 | 0.5419527 | 35 | 100% | 35 | 100% |
| p__Firmicutes;c__Bacilli;o__Lactobacillales;f__Lactobacillaceae;g__Lactobacillus | 0.0461094 | 0.0367840 | 35 | 100% | 35 | 100% |
| p__Firmicutes;c__Clostridia;o__Clostridiales;f__Lachnospiraceae;g__Blautia | 0.0076647 | 0.0080936 | 35 | 100% | 35 | 100% |
| p__Firmicutes;c__Clostridia;o__Clostridiales;f__Lachnospiraceae;g__NA | 0.3021298 | 0.2161252 | 35 | 100% | 35 | 100% |
| p__Firmicutes;c__Clostridia;o__Clostridiales;f__Ruminococcaceae;g__NA | 0.1915469 | 0.0801266 | 35 | 100% | 35 | 100% |
| p__Firmicutes;c__Clostridia;o__Clostridiales;f__Lachnospiraceae;g__Lachnoclostridium | 0.0241465 | 0.0066197 | 35 | 100% | 34 | 97% |
| p__Firmicutes;c__Clostridia;o__Clostridiales;f__Lachnospiraceae;g__Marvinbryantia | 0.0102844 | 0.0020584 | 35 | 100% | 34 | 97% |
| p__Firmicutes;c__Clostridia;o__Clostridiales;f__Ruminococcaceae;g__Oscillibacter | 0.0178420 | 0.0128484 | 35 | 100% | 34 | 97% |
| p__Firmicutes;c__Clostridia;o__Clostridiales;f__Ruminococcaceae;g__Anaerotruncus | 0.0091671 | 0.0028712 | 35 | 100% | 33 | 94% |
| p__Firmicutes;c__Erysipelotrichia;o__Erysipelotrichales;f__Erysipelotrichaceae; g__Turicibacter | 0.0312716 | 0.0040361 | 35 | 100% | 9 | 26% |
| p__Proteobacteria;c__Betaproteobacteria;o__Burkholderiales;f__Alcaligenaceae; g__Parasutterella | 0.0035292 | 0.0033895 | 34 | 97% | 33 | 97% |
| p__Firmicutes;c__Clostridia;o__Clostridiales;f__Ruminococcaceae;g__Ruminiclostridium | 0.0048165 | 0.0021194 | 34 | 97% | 28 | 82% |
| p__Firmicutes;c__Clostridia;o__Clostridiales;f__NA;g__NA | 0.0015219 | 0.0159433 | 33 | 94% | 33 | 100% |
| p__Firmicutes;c__Erysipelotrichia;o__Erysipelotrichales;f__Erysipelotrichaceae; g__Erysipelatoclostridium | 0.0034674 | 0.0011575 | 33 | 94% | 13 | 39% |
| p__Firmicutes;c__Clostridia;o__Clostridiales;f__FamilyXIII;g__NA | 0.0008595 | 0.0007346 | 32 | 91% | 25 | 78% |
| p__Firmicutes;c__Erysipelotrichia;o__Erysipelotrichales;f__Erysipelotrichaceae;g__NA | 0.0019808 | 0.0014375 | 31 | 89% | 22 | 71% |
| p__Firmicutes;c__Clostridia;o__Clostridiales;f__Clostridiaceae;g__Clostridium | 0.0049239 | 0.0001280 | 31 | 89% | 2 | 6% |
| p__Firmicutes;c__Clostridia;o__Clostridiales;f__Lachnospiraceae;g__Acetatifactor | 0.0015409 | 0.0014789 | 30 | 86% | 24 | 80% |
| p__Firmicutes;c__Clostridia;o__Clostridiales;f__Peptococcaceae;g__NA | 0.0008391 | 0.0003786 | 30 | 86% | 18 | 60% |
| p__Tenericutes;c__Mollicutes;o__NA;f__NA;g__NA | 0.0014253 | 0.0013153 | 29 | 83% | 19 | 66% |
| p__Firmicutes;c__Clostridia;o__Clostridiales;f__Lachnospiraceae;g__Roseburia | 0.0017629 | 0.0003895 | 28 | 80% | 10 | 36% |
| p__Firmicutes;c__Clostridia;o__Clostridiales;f__Peptostreptococcaceae;g__Romboutsia | 0.0017179 | 0.0001376 | 27 | 77% | 1 | 4% |
| p__Verrucomicrobia;c__Verrucomicrobiae;o__Verrucomicrobiales; f__Verrucomicrobiaceae;g__Akkermansia | 0.0058049 | 0.0074285 | 26 | 74% | 8 | 31% |
| p__Actinobacteria;c__Coriobacteriia;o__Coriobacteriales;f__Coriobacteriaceae; g__Enterorhabdus | 0.0017016 | 0.0008129 | 25 | 71% | 22 | 88% |
| p__Actinobacteria;c__Coriobacteriia;o__Coriobacteriales;f__Coriobacteriaceae; g__Adlercreutzia-Asaccharobacter | 0.0007613 | 0.0003091 | 25 | 71% | 12 | 48% |
| p__Firmicutes;c__Clostridia;o__Clostridiales;f__Lachnospiraceae;g__Tyzzerella | 0.0004368 | 0.0004131 | 24 | 69% | 15 | 63% |
| p__Firmicutes;c__Clostridia;o__Clostridiales;f__Christensenellaceae;g__NA | 0.0005363 | 0.0002060 | 20 | 57% | 12 | 60% |
| p__Bacteroidetes;c__Bacteroidia;o__Bacteroidales;f__Bacteroidaceae;g__Bacteroides | 0.0088374 | 0.0217698 | 19 | 54% | 19 | 100% |
| p__Firmicutes;c__Clostridia;o__Clostridiales;f__Lachnospiraceae;g__Acetitomaculum | 0.0005895 | 0.0000182 | 16 | 46% | 1 | 6% |
| p__Actinobacteria;c__Actinobacteria;o__Bifidobacteriales;f__Bifidobacteriaceae; g__Bifidobacterium | 0.0052494 | 0.0192585 | 14 | 40% | 13 | 93% |
| p__Tenericutes;c__Mollicutes;o__Anaeroplasmatales;f__Anaeroplasmataceae; g__Anaeroplasma | 0.0004937 | 0.0050494 | 13 | 37% | 11 | 85% |
| p__Proteobacteria;c__Betaproteobacteria;o__Burkholderiales;f__Burkholderiaceae; g__Burkholderia-Burkholderia-Paraburkholderia | 0.0002088 | 0.0000000 | 13 | 37% | 0 | 0% |
| p__Actinobacteria;c__Coriobacteriia;o__Coriobacteriales;f__Coriobacteriaceae;g__NA | 0.0162395 | 0.0000237 | 11 | 31% | 1 | 9% |
| p__Actinobacteria;c__Coriobacteriia;o__Coriobacteriales;f__Coriobacteriaceae; g__Parvibacter | 0.0004222 | 0.0000879 | 6 | 17% | 1 | 17% |
| p__Firmicutes;c__Erysipelotrichia;o__Erysipelotrichales;f__Erysipelotrichaceae; g__Allobaculum | 0.0007505 | 0.0033967 | 5 | 14% | 2 | 40% |
| p__Proteobacteria;c__Gammaproteobacteria;o__Pseudomonadales;f__Moraxellaceae; g__Acinetobacter | 0.0000623 | 0.0001330 | 3 | 9% | 0 | 0% |
| p__Bacteroidetes;c__Bacteroidia;o__Bacteroidales;f__Porphyromonadaceae; g__Odoribacter | 0.0001098 | 0.0007406 | 2 | 6% | 1 | 50% |
| p__Actinobacteria;c__Coriobacteriia;o__Coriobacteriales;f__Coriobacteriaceae; g__Adlercreutzia | 0.0000392 | 0.0000000 | 1 | 3% | 0 | 0% |
| p__Firmicutes;c__Bacilli;o__Lactobacillales;f__Enterococcaceae;g__Enterococcus | 0.0000259 | 0.0000275 | 1 | 3% | 0 | 0% |
| p__Firmicutes;c__Clostridia;o__Clostridiales;f__Ruminococcaceae;g__Intestinimonas | 0.0000094 | 0.0000000 | 1 | 3% | 0 | 0% |
| p__Firmicutes;c__Clostridia;o__Clostridiales;f__Ruminococcaceae;g__Oscillibacter-Oscillospira | 0.0000142 | 0.0000125 | 1 | 3% | 0 | 0% |
| p__Proteobacteria;c__Alphaproteobacteria;o__Rhodospirillales;f__Rhodospirillaceae; g__Azospirillum | 0.0000000 | 0.0000187 | 0 | 0% | 0 | N/A |
| p__Proteobacteria;c__Gammaproteobacteria;o__Enterobacteriales;f__Enterobacteriaceae; g__Enterobacter | 0.0000000 | 0.0000125 | 0 | 0% | 0 | N/A |
| p__Proteobacteria;c__Gammaproteobacteria;o__Enterobacteriales;f__Enterobacteriaceae; g__Escherichia-Shigella | 0.0000000 | 0.0000273 | 0 | 0% | 0 | N/A |

N= 35 pairs donor-recipient pairs.
