# Supplementary material for: Fecal microbiota transplanted from old mice promotes more colonic inflammation, proliferation, and tumor formation in azoxymethane-treated A/J mice than microbiota originating from young mice
Source: Gut Microbes. 2023 Nov 29;15(2):2288187. doi: 10.1080/19490976.2023.2288187 (PMC10730208; doi:10.1080/19490976.2023.2288187)
Supplement: Figure S3. FMT transplant efficiency.docx [file KGMI_A_2288187_SM6853.docx]

**Figure S4. Cosine similarity analysis of donors and recipients**

DY to RY

DO to RO

FMT efficiency measured by cosine similarity between matched donors and recipients. No significant difference between groups (P=0.22). Combined similarity for all donor-recipient pairs = 0.83 ± 0.02 (Range = 0.63 – 0.99). DO, Old Donors; DY, Young Donors; RO, Recipients of Old Donor; RY, Recipients of Young Donors. N= 35 pairs.
